# Effects of a physical fitness program on memory and blood viscosity in sedentary elderly men

**DOI:** 10.1590/1414-431X20154529

**Published:** 2015-07-28

**Authors:** H.K. Antunes, M.T. De Mello, R.F. Santos-Galduróz, J.C.F. Galduróz, V.Aquino Lemos, S. Tufik, O.F.A. Bueno

**Affiliations:** 1 Departamento de Biociências, Universidade Federal de São Paulo, Santos, SP, Brasil; 2 Departamento de Psicobiologia, Universidade Federal de São Paulo, São Paulo, SP, Brasil; 3 Centro de Estudos em Psicobiologia e Exercício, São Paulo, SP, Brasil; 4 Centro de Matemática, Computação e Cognição, Universidade Federal do ABC, Santo André, SP, Brasil; 5 Departamento de Esportes, Faculdade de Educação Fisica, Fisioterapia e Terapia Ocupacional, Universidade Federal de Minas Gerais, Belo Horizonte, MG, Brasil

**Keywords:** Cognition, Elderly adults, Physical exercise

## Abstract

The aim of this study was to investigate the effects of a 6-month exercise program on cognitive function and blood viscosity in sedentary elderly men. Forty-six healthy inactive men, aged 60–75 years were randomly distributed into a control group (n=23) and an experimental group (n=23). Participants underwent blood analysis and physical and memory evaluation, before and after the 6-month program of physical exercise. The control group was instructed not to alter its everyday activities; the experimental group took part in the fitness program. The program was conducted using a cycle ergometer, 3 times per week on alternate days, with intensity and volume individualized at ventilatory threshold 1. Sessions were continuous and maximum duration was 60 min each. There was significant improvement in memory (21%; P<0.05), decreased blood viscosity (−19%; P<0.05), and higher aerobic capacity (48%; P<0.05) among participants in the experimental group compared with the control group. These data suggest that taking part in an aerobic physical fitness program at an intensity corresponding to ventilatory threshold-1 may be considered a nonmedication alternative to improve physical and cognitive function.

## Introduction

Aging is a dynamic and progressive process that involves morphological, functional, biochemical and psychological transitions (1). This process is often accompanied by impairment of cognitive functions such as memory, attention, visuospatial ability and information processing speed (2). An increment in blood viscosity is also common with aging (3). Moreover, some authors have observed that reductions in cerebral blood flow have been linked to reduced cognitive function in older people and patients with Alzheimer’s disease (4). One study demonstrated a significant correlation between decreased cognitive function and increased blood viscosity, suggesting that high blood viscosity is an important factor in cognition to be investigated in future studies (5). Poor cognitive function has been associated with development of comorbid disease, increased risk of dementia, loss of independence, hospitalization, institutionalization, and death (6,7). These alterations affect quality of life in the elderly population, particularly by restricting their social life and gradually reducing their independence.

Physical exercise has received much attention as a potential protective factor for neurocognitive functioning and as an alternative to medication for reducing blood viscosity (8). Exercise is thought to contribute to improving cognitive performance as a result of the cumulative physical and psychological changes that occur over the course of multiple exercise training sessions, particularly with aerobic training (9). A study by Dik et al. (10) showed that physical exercise can mitigate changes in cognitive functions. Some authors have shown that physical activity is inversely associated with cognitive decline in elderly adults. Another study reported a close correlation between improved physical fitness and aerobic capacity and better cognitive functioning (11).

However, some studies failed to find a significant association between cognitive functioning and aerobic exercise training (12). Conflicting data in the literature raise some questions as to the real effect of physical exercise on cognitive functions. Despite controversy, epidemiological studies have confirmed that moderately active people have less risk of mental impairment than those who are sedentary. The research also supports that taking part in physical exercises programs leads to physical and neuropsychological benefits (13) and physically active individuals are likely to have faster cognitive processing speeds (8).

Given that few and varied randomized interventions have examined the effects of aerobic training on cognition, and few studies have managed to advance understanding of the mechanisms involved, important questions remain in the literature. We hypothesized that aerobic exercise training in elderly adults could: promote a decrease in blood viscosity by improving aerobic capacity, improve blood circulation in the brain, and have a positive impact on cognition. Thus, the aim of this study was to examine the impact of a 6-month physical exercise program on cognitive function and blood viscosity in sedentary elderly adults.

## Material and Methods

### Participants

Forty-six healthy sedentary male volunteers were selected and randomly distributed into two groups: a control group (n=23; age=65.86±3.80 years; weight=76.38±11.10 kg; height=1.67±0.58 m; body mass index=27.17±3.09 kg/m^2^) and an experimental group (n=23; age=68.08±5.49 years; weight=77.56±13.45 kg; height=1.69±0.85 m; body mass index=27.06±3.75 kg/m^2^). We included participants with at least 7 years of formal education who were nonsmokers and had a sedentary lifestyle (i.e., no habitual physical activity), no clinical symptoms or indicators of cardiovascular disease, no medication that could alter cardiovascular or cognitive function, no psychotropic drug use or of any pharmaceutical drug for which physical activity is a contraindication or that may negatively influence cognitive function, and no recent surgical intervention (in the past 6 months). The criterion used to determine a sedentary lifestyle was gathered from three sources of information: an interview, a short questionnaire measuring regular physical activity (14), and analysis of oxygen uptake (<25 mL·kg^−1^·min^−1^). Participants were given resting and exercise electrocardiograms to assess cardiovascular health.

The Mini Mental State Examination (MMSE) was also administered (15), divided into five subtests (orientation, immediate memory, attention and calculation, recall and language). To better describe sample demographical variables, the cutoff score was set to 24 points. We also used Raven’s Standard Progressive Matrices sets A-E to evaluate general intelligence and confirm that participants had no signs of cognitive deficits greater than those expected for their age (16).

All methods and procedures were approved by the Research Ethics Committee of the Universidade Federal de São Paulo/Hospital São Paulo (#207/01) and were in accordance with the principles of the Helsinki Declaration of 1975. The nature of the study, its aims and possible risks were carefully explained to participants in advance, and all participants signed consent forms.

### Description of groups

The experimental group took part in an aerobic physical fitness program 3 times a week on alternate days for 6 months. Sessions were continuous and lasted 60 min; the initial 20-min session was gradually increased to the maximum 60 min. A LifeCycle 9500HR cycle ergometer (Life Fitness, USA) was used for all sessions. Participants underwent prior ergospirometric evaluation at ventilatory threshold 1 (VT-1). Exercise intensity was prescribed in accordance with the concept of an anaerobic threshold proposed by Wasserman and McIlroy (17). Participants’ heart rate was monitored during sessions. Stretching and joint flexibility exercises were included as supplementary activities.

Participants in the control group were asked not to vary their everyday activities or to begin any type of physical fitness program. Volunteers were monitored longitudinally through monthly phone calls to maintain contact and to keep them informed of the study’s progress. Participants in the control group were also informed that although they could not currently take part in the fitness program, they could do so after the intervention period had ended.

### Experimental procedure

#### Ergospirometric test

Cardiopulmonary assessment was carried out, in which exhaled gases were analyzed by direct measurement of oxygen consumption to determine ventilatory threshold. Tests were conducted using a Vmax 29 series metabolic cart (SensorMedics, USA). The system was pre-test calibrated using known gas concentrations (O_2_ and CO_2_), and flow sensor calibration was carried out with a 3-L syringe. A flow-through face mask (Hans Rudolph Inc. USA) was positioned on each participant before testing. The procedure involved 25-W load increments every 2 min; initial warm-up load was 3 min at 25 W and the test was terminated on reaching the safety margin for peak oxygen consumption. Blood pressure was monitored during testing with a manual sphygmomanometer and heart rate monitored with a Polar Vantage NV device (Polar Electro, Finland). To avoid circadian interference, tests were conducted at the same time each day (8:00–11:00 am) in a climate-controlled standard laboratory environment. Ventilatory threshold 1 (VT-1) was assessed by two blinded independent investigators, and the criteria used to determine oxygen consumption at VT-1 were as described by Wasserman et al. (18) and Wasserman and Koike (19).

All evaluations, including blood analysis and physical and memory assessments, were performed 48 h before the start and 48 h after the end of the 6-month program, following the same procedure. The testing sequence was planned to avoid interference between tests as much as possible, according to Spreen and Strauss (20). The tests administered are described below.

### Neuropsychological assessment

#### Picture Arrangement

(WAIS-III; Wechsler Adult Intelligence Scale, 3rd edition) In this subtest, individuals are presented with a series of cards in an incorrect order and asked to arrange the pictures in the correct order to tell a story that makes sense. This task provides information about an individual’s nonverbal understanding of social interaction and reasoning abilities; performance is related to the ability to understand precursors and consequences of events, and is a measure of planning ability (21).

#### Corsi Block-tapping

This test is a measure of attention, immediate memory capacity, and spatial memory. The apparatus used consists of a set of nine blocks arranged irregularly on a wooden board. The blocks are tapped by the examiner at the rate of one block per second in novel sequences of increasing length. In this study, the subject was required to reproduce each block-tapping sequence immediately after the examiner, in direct (forward) and inverse (backward) order (22).

#### Verbal Paired Associates

This test consists of a list of eight paired words. Individuals are required to learn four pairs of semantically related words considered “easy” (for example, rose-flower) and four pairs of unrelated words considered “hard” (for example, hot-quiet) across three study test trials, followed after 30 min by a delayed recall test. This task gives information about memory acquisition capacity and yields a memory acquisition score, a learning score, as well as delayed recall and recognition scores. Thus, this task is sensitive to diverse aspects of memory functioning (23).

#### Free Word Recall

This test evaluates verbal episodic declarative memory and provides evidence of short- and long-term memory function independent of the central executive processes related to capacity to integrate information. In this test, individuals are presented with 12 lists, numbered 1–12, each containing 15 common Portuguese disyllabic and trisyllabic words. For even-numbered lists, positions 7, 8, and 9 contained semantically related words (e.g., fire, firemen). After the examiner slowly presents each list (one at a time), individuals are asked to verbally recall as many words as possible, in any order. There is no time limit. After recall, a new list is presented, and so on, until the 12 lists have been presented. The presentation order of stimulus words influences recall, causing an increased or decreased probability of recall according to the position of the word on the list (24).

### Blood assessment

A blood sample (4 mL) for hemorheological measurements was drawn from each participant into a Vacutainer evacuation tube (Becton, Dickinson and Company, USA) containing potassium EDTA anticoagulant. Whole blood viscosity was determined with a DV-III model Wells-Brookfield Cone/Plate Viscometer (Brookfield Engineering Labs. Inc., USA), following the technique standardized by Galduróz et al. (25). The temperature of the viscometer was maintained at 37±0.3°C, and viscometric measurements were taken at shear rate 250/s.

### Statistical analysis

STATISTICA 12 (StatSoft, USA) software for Windows was used for statistical analysis. The Shapiro-Wilk test was applied to determine whether the distribution curve was normal. Data are reported as means±SD. To determine the effect of intervention periods (time effect), we used two-way repeated measures ANOVA with a Duncan *post hoc* test. Student’s *t*-test was used to determine significant differences between the control and experimental groups in recall probability for word recall testing. The minimum significance level was set to 0.05 (5%).

## Results

Before initiating the experimental protocol, we observed no significant differences between the study groups, suggesting that the participants had similar conditions with respect to age (t=1.47; P=0.14), weight (t=0.14; P=0.88), height (t=0.61; P=0.53) and body mass index (t=−0.25; P=0.79).

Table 1 shows the results of neuropsychological assessment. The post-training data revealed the strongest effect in the picture arrangement test (F=43.16; P<0.001), with the experimental group showing an increased score when compared with baseline scores (P<0.01) and the control group (P<0.04). Similar results were observed for the Corsi Block-tapping Task (F=11.18; P<0.001) with improvement in memory and attention seen in the forward task, compared with the baseline condition (P<0.04) and control group (P<0.008). Comparing baseline and post-training results, the experimental group also showed improved memory acquisition capacity, as measured by the Verbal Paired Associates test. Post-training scores for “hard” word pairs, after one (F=5.18; P<0.04); two (F=5.63; P<0.02) and three trials (F=4.48; P<0.04), were increased compared with baseline and control group scores (P<0.05, for all). For Free Word Recall, better recall of non-semantically related (F=18.33; P<0.04) and semantically related words (F=26.09; P<0.001), as well as fewer intrusions (F=8.70; P<0.05), was observed in the experimental group compared with the baseline condition and control group (P<0.05, for all). No changes were seen for other variables.

**Table:**
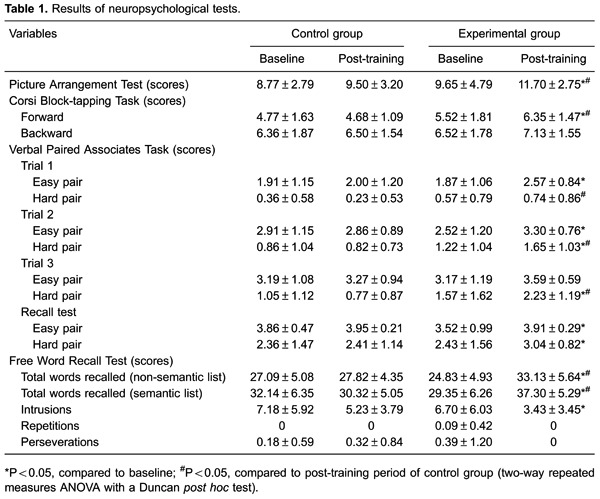

**Figure 1:**
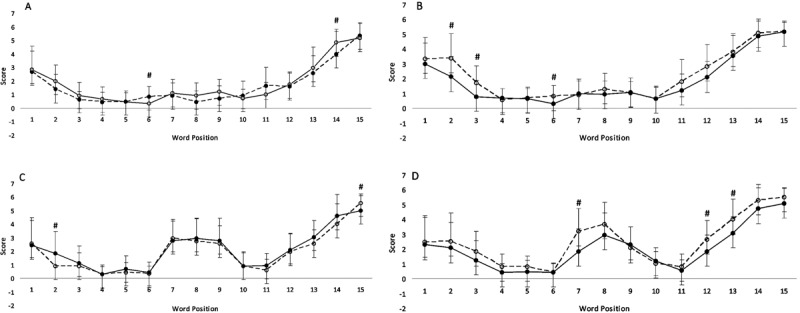
Serial curve position of Free Word Recall test. Assessments were made at baseline and post-training. *A*: Lists with no semantically related words, at baseline; *B*: lists with no semantically related words, post-training; *C*: lists with semantically related words, at baseline; *D*: lists with semantically related words, post-training. The scores of each position are reported as means±SD. ^#^P≤0.05 compared to control group (Student’s *t*-test for independent samples).

Figure 1 shows a serial position curve for the experimental and control groups on the Free Word Recall test. At baseline, for lists that contained no semantically related words (Figure 1A), differences were observed in words at positions 6 (t=3.18; P=0.002) and 14 (t=-2.48; P=0.01), with the experimental group showing lower and higher recall, respectively, compared with the control group. For lists containing semantically related words (Figure 1C), differences were seen for words in positions 2 (t=-2.23; P=0.03) and 15 (t=2.02; P=0.04), with the experimental group presenting lower and higher recall, respectively, compared with controls. Despite these differences, it is possible to affirm that both groups had homogenous pre-intervention skills. In Figure 1A and C, both groups presented U-shaped curves, indicating a primacy effect and recency effect related to short-term memory. In Figure 1C, both groups demonstrated a W-shaped curve, indicating recall of semantically related words in the middle list positions.

After the intervention period, for lists with no semantically related words (Figure 1B), the experimental group shows improvement in positions 2 (t=2.62; P=0.01), 3 (t=2.81; P=0.007) and 6 (t=2.78; P=0.07). For lists with semantically related words (D), improvement was noted in positions 7 (t=2.79; P=0.007), 12 (t=2.05; P=0.04) and 13 (t=2.29; P=0.02). These data revealed an improvement in primacy and recency effects, respectively.

**Figure 2:**
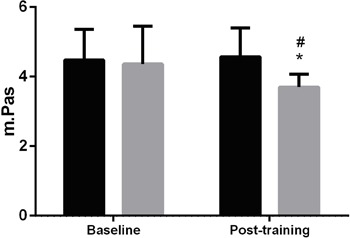
Blood viscosity. Control group: black bars; Experimental group: gray bars. Data are reported as means±SD. *P≤0.05 compared with baseline; ^#^P≤0.05 compared to control group (two-way repeated measures ANOVA with a Duncan *post hoc* test).

Figure 2 shows the results of blood viscosity testing. A significant difference was seen in this parameter when comparing groups before and after intervention (F=9.25; P<0.003). The experimental group showed a decrease in blood viscosity compared with the baseline condition (P<0.0001) and the control group (P<0.009).

**Figure 3:**
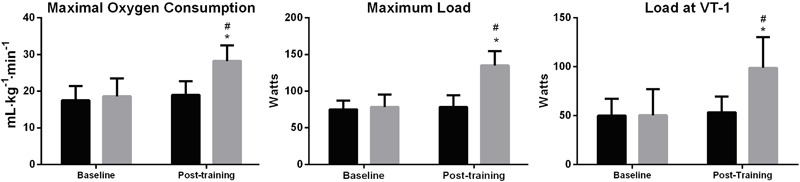
Parameters of physical fitness testing. Control group: black bars; Experimental group: gray bars. Data are reported as means±SD. *P≤0.05 compared to baseline; ^#^P≤0.05 compared to control group (two-way repeated measures ANOVA with a Duncan *post hoc* test).

Finally, Figure 3 shows the results of physical fitness testing. A significant improvement in physical capacity was demonstrated by increased maximal oxygen consumption (F=80.10; P<0.002) compared with the baseline (P<0.003) and controls (P<0.006). Similar results were observed in maximum load (F=64.41; P=0.004), and load at VT-1 intensity (F=70.87; P<0.0001), with the experimental group showing improvement compared with the baseline condition (P<0.00006) and control group (P<0.0001).

## Discussion

In this study, we applied a program of physical exercise at VT-1 intensity, 3 times a week for 6 months and observed improvements in cognitive functioning in the experimental group compared with the control group. In general, we observed a marked enhancement of planning and reasoning ability, executive functions (picture arrangement); attention, praxia, spatial memory (Corsi Block-tapping Task); verbal memory, learning, and capacity to integrate information (Free Word Recall and Verbal Paired Associates).

These data are in agreement with several previous reports of improved cognitive functioning in elderly adults who engage in regular physical exercise (26). Several mechanisms could explain the improvement observed in cognition, such as improved cerebral blood flow. Elderly adults with cognitive impairment have demonstrated lower cerebral blood flow in the left frontal and temporal cortices as well as in the bilateral medial frontal and anterior cingulate cortices. These brain areas with low blood flow are suggestive of a mechanism underlying cognitive impairment (27). It is possible that physical exercise enhances physiological stimulation, contributing to neurotransmitter release and increased blood flow, which in turn promote improved cognitive performance (28).

Increases in neurotrophic stimulation (including hippocampal neurogenesis, which steadily declines with age) are associated with cognitive functioning. The very low levels of hippocampal neurogenesis persisting in the aged brain are suspected as underlying the cognitive deficits observed in elderly populations (29). Studies have suggested that physical exercise may stimulate nerve cell growth in adult mice (30), If these studies were to be confirmed in humans, this would indicate that physical activity might provide a neuronal buffer to protect against age-related neurodegeneration.

Another perspective is related to brain-derived neurotrophic factor (BDNF), essential for the development, increase, survival, and synaptic development of new neurons. BDNF supports more efficient brain plastic and adaptive processes, which can influence and improve cognitive functioning in older adults (31,32). Positive impacts of physical exercise on improved BDNF levels and cognitive functioning have been reported. Despite controversial findings, BDNF is considered one of the most plausible factors involved in cognitive benefits associated with physical activity (33).

Improvements in physical fitness in the experimental group, evident from improved parameters for oxygen consumption, maximum load and higher load at VT-1 intensity, reflect their improved aerobic capacity. This improvement may explain the beneficial effects on cognitive performance obtained by participants in this group. Studies, such as by et al. (34), have suggested that physical exercise maintains vascular integrity and improves the flow of oxygen to the brain, thus making cognitive processing faster and more efficient in physically active persons, owing to mechanisms like better cerebral circulation and alteration in the synthesis and degradation of neurotransmitters.

We observed a decrease in blood viscosity among participants in the experimental group. This decrease may be related to the improvements in cognitive functions seen in that group. We believe that the fitness program served to promote improvement in cardiovascular functioning and enhance cerebral blood flow. Although the actual mechanism remains unclear, our hypothesis is that physical exercise increases fluid transfer from the blood to the interstitial space. Another possible mechanism is related to the increase in erythrocyte volume caused by exercise. The accompanying rise in plasma volume would result in decreased hematocrit, which is associated with lower blood viscosity. Furthermore, some authors have described blood viscosity as a function of plasma hematocrit and erythrocyte deformability, and that physical exercise is capable of improving this deformability (35).

An important implication of engaging in regular physical exercise is that it may be an effective method to maintain functional skills and promote well-being in elderly adults (32). In addition, exercise is relatively low-cost and is available to large numbers of people. Varying the intensity of physical exercise in relation to VT-1 leads to beneficial alteration of some physiological parameters. More intensive exercise leads to a better response, suggesting that the adaptive response may be intensity-dependent. According to Angevaren et al. (36), the intensity and variation of weekly physical activities are positively and significantly associated with cognitive performance and overall cognitive functioning. In contrast, a recently published article by our group showed that moderate aerobic exercise improved various cognitive parameters in an elderly population, including sustained attention, mental control, verbal comprehension, visual and spatial orientation, capacity planning and organization, response speed and executive functions (37).

A limitation of our study is that our sample comprised healthy elderly males with excellent physical and cognitive status. This situation might cause difficulty in establishing cognitive and blood viscosity improvements.

Our sample included sedentary elderly males. It is known that physical inactivity is associated with elevated mortality risk in middle-aged and older adults (38) and is considered the largest public health problem of the 21st century (39). Long-term, regular physical activity can reduce morbidity and mortality, postpone disability, prolong life, and can potentially counterbalance some of the negative effects of aging. It is important to consider that physical exercise may not have a meaningful impact on cognition when it is undertaken infrequently. Long-term, regular physical activity may be a useful intervention contributing to improvements in cognitive performance, as the result of cumulative physical and psychological changes during the course of multiple exercise training sessions (40).

Our data suggest that a 6-month program of aerobic exercise at VT-1 intensity may be an effective alternative to medication for cognitive and physical improvement in elderly adults without dementia. These data contribute to understanding of the relationship between exercise and cognition; however, many questions remain. Improvement in cognition caused by aerobic exercise remains open to debate, thus, further studies are needed to clearly understand the mechanisms involved.
